# Six new species of *Cichlidogyrus* Paperna, 1960 (Platyhelminthes: Monogenea) from the gills of cichlids (Teleostei: Cichliformes) from the Lomami River Basin (DRC: Middle Congo)

**DOI:** 10.1186/s13071-020-3927-4

**Published:** 2020-04-09

**Authors:** Mare Geraerts, Fidel Muterezi Bukinga, Maarten P. M. Vanhove, Antoine Pariselle, Auguste Chocha Manda, Emmanuel Vreven, Tine Huyse, Tom Artois

**Affiliations:** 1 Research Group Zoology: Biodiversity and Toxicology, Centre for Environmental Sciences, Hasselt University, Diepenbeek, Belgium; 2 Section de Parasitologie, Département de Biologie, Centre de Recherche en Hydrobiologie, Uvira, Democratic Republic of the Congo; 3 Zoology Unit, Finnish Museum of Natural History, University of Helsinki, Helsinki, Finland; 4 Department of Botany and Zoology, Faculty of Science, Masaryk University, Brno, Czech Republic; 5 Laboratory of Biodiversity and Evolutionary Genomics, Department of Biology, University of Leuven, Leuven, Belgium; 6 ISEM, Université de Montpellier, CNRS, IRD, EPHE, CIRAD, INRAP, Montpellier, France; 7 Laboratory Biodiversity, Ecology and Genome. Research Centre Plant and Microbial Biotechnology, Biodiversity and Environment, Faculty of Sciences, Mohammed V University, Rabat, Morocco; 8 Unité de recherche en Biodiversité et Exploitation durable des Zones Humides (BEZHU), Faculté des Sciences Agronomiques, Université de Lubumbashi, Lubumbashi, Democratic Republic of the Congo; 9 Ichthyology Section, Zoology Department, Royal Museum of Central Africa, Tervuren, Belgium; 10 Department of Biology, Royal Museum of Central Africa, Tervuren, Belgium

**Keywords:** Africa, DRC, Cichlidae, Monogenea, *Cichlidogyrus*, *Serranochromis*, *Tilapia*, *Orthochromis*, Diversity

## Abstract

**Background:**

Monogenea van Beneden, 1858 is a group of parasitic flatworms, commonly found infecting bony fish. Several genera, such as *Cichlidogyrus* Paperna, 1960, are reported to include potential pathogenic species that can negatively impact aquaculture fish stocks. They can switch from introduced to native fish and vice versa. In Africa (and all over the world), fish species belonging to Cichlidae are often kept in aquaculture and represent a major source of food. Thus, research on the biodiversity and occurrence of monogenean species on these fish is of importance for aquaculture and conservation. The present study is a survey of the diversity of species of *Cichlidogyrus* in the south of the Democratic Republic of the Congo (DRC) on three cichlid species: *Orthochromis* sp. ‘Lomami’, *Serranochromis* cf. *macrocephalus*, and *Tilapia sparrmanii* Smith, 1840.

**Methods:**

Specimens of *Cichlidogyrus* were isolated from the gills and mounted on glass slides with Hoyer’s medium. The genital and haptoral hard parts were measured and drawn using interference contrast.

**Results:**

In total, six species of *Cichlidogyrus* were found, all new to science: *C. bulbophallus* n. sp. and *C. pseudozambezensis* n. sp. on *S.* cf. *macrocephalus*, *C. flagellum* n. sp. and *C. lobus* n. sp. on *T. sparrmanii*, *C. ranula* n. sp. on *S.* cf. *macrocephalus* and *Orthochromis* sp. ‘Lomami’, and *C. maeander* n. sp. found on *Orthochromis* sp. ‘Lomami’ and *T. sparrmanii*. The first four species are considered to be strict specialists, *C. ranula* n. sp. an intermediate generalist and *C. maeander* n. sp. a generalist. These parasite species show morphological similarities to species found in the Lower Guinea and Zambezi ichthyofaunal provinces, which might be explained by past river capture events between river systems of the Congo Province and both these regions.

**Conclusions:**

*Serranochromis* cf. *macrocephalus* and *Orthochromis* sp. ‘Lomami’ can harbour respectively three and two species of *Cichlidogyrus*, all described in this study. *Tilapia sparrmanii* can harbour seven species, of which three are described in the present study. These results highlight the species diversity of this parasite genus in the Congo Basin.

## Background

Since the second half of the 20th century, the number of described monogenean species increased substantially, as interest in fish parasites increased in general because of intensified aquaculture and stocking [1, 2]. At the moment, more than 5500 species and 750 genera have been described [3, 4]. Within the Monogenea, the Gyrodactylidae van Beneden & Hesse, 1863 and the Dactylogyridae Bychowsky, 1933 are the most species-rich families, each consisting of more than 500 to thousands of species described [2, 3].

Several genera of the Monogenea, such as *Cichlidogyrus* Paperna, 1960, *Dactylogyrus* Diesing, 1850 and *Gyrodactylus* von Nordmann, 1832, are reported to include potential fish pathogens, especially in aquaculture stocks [3–5]. There is a possibility that they switch from introduced to native fish, where they can cause high mortality (see [5–7] and references herein). Additionally, spillback phenomena from native to introduced fish have been observed [8]. Thus, research on the biodiversity and occurrence of monogenean species is of importance for aquaculture and conservation, especially in a time of intense aquaculture and worldwide translocation of their hosts.

In Africa (and all over the world), fish species belonging to the Cichlidae are often kept in aquaculture and represent a major source of food [9]. Among the non-ostariophysan groups, the Cichlidae is the most species-rich family, with about 1700 described species belonging to 250 genera. More than 1100 of these species occur in the inland waters of Africa [10–12]. At present, African cichlid fishes are known to harbour six genera of Monogenea: two of them are mesoparasites living in the host’s body cavity and four are ectoparasites found on the gills of their host [13]. The gill parasites include three genera belonging to the Dactylogyridae (*Cichlidogyrus* [14], *Onchobdella* Paperna, 1968 [15] and *Scutogyrus* Pariselle & Euzet, 1995 [16]) and one genus belonging to the Gyrodactylidae (*Gyrodactylus*) [17]. Among these, *Cichlidogyrus* is the most species-rich, including 125 described species [18–20]. Species of this genus naturally occur on cichlids from Africa and the Middle East [21, 22].

The present study is a survey of the diversity of species of *Cichlidogyrus* on cichlid species in the south of the Democratic Republic of the Congo (DRC). The diversity of this parasite genus is investigated for the fish hosts *Orthochromis* sp. ‘Lomami’ and *Serranochromis* cf. *macrocephalus* from the upper course of the Lomami River, and *Tilapia sparrmanii* Smith, 1840 from the Ngulungu River, an affluent of the upper course of the Lomami River (DRC). These three fish species belong to the so-called haplotilapiine lineage [23–25]. *Tilapia sparrmanii* belongs to the tribe Tilapiini which, together with the tribe Steatocranini, form the sister taxon to the group comprising the East African radiations. The latter contains *Orthochromis* Greenwood and *Serranochromis* Regan. *Orthochromis* is a polyphyletic genus consisting of haplochromine cichlid species inhabiting exclusively riverine habitats [23]. One monophyletic taxon occurs in the Malagarasi River system (suggested by Salzburger et al. [23] to be named *Schwetzochromis*), the other taxon in the Congo River system. The taxon occurring in the Congo River system falls in a cluster consisting of two main monophyletic clades: one clade including species of *Orthochromis*, the other including *Serranochromis* [23].

The Lomami River is a left bank affluent of the Middle Congo, running through the Upper Congo ecoregion [26]. For most of its course it runs parallel to the Upper Congo (Lualaba), joining the Middle Congo downstream from Kisangani, near Yangambi. The species richness of fish in the Congo Basin is largest in the main courses of the major rivers [27]. However, a relatively low number of fish species are reported from the Lomami River, which is most likely an indication of limited inventorying in this river [27]. By consequence, the knowledge on the diversity of monogenean fish parasites in this river is also non-existent, despite the aforementioned conservational and economic importance of their cichlid hosts [2]. Therefore, additional sampling in this river is needed to better understand the true diversity of fish and their parasites [27].

To date, only few studies have reported gill parasites of *Serranochromis macrocephalus* (Boulenger), *T. sparrmanii* or species of *Orthochromis*. Five species of *Cichlidogyrus* have been reported from *S. macrocephalus*: *C. dossoui* Douëllou, 1993 [28]; *C. halli* (Price & Kirk, 1967) [28]; *C. quaestio* Douëllou, 1993 [28]; *C. sclerosus* Paperna & Thurston, 1969 [28]; and *C. zambezensis* Douëllou, 1993 [28, 29]. Four species of *Cichlidogyrus* has been reported from *T. sparrmanii* (*C. dossoui* [30]; *C. papernastrema* Price, Peebles & Bamford, 1969 [29, 31]; *C. quaestio* [30]; and *C. tiberianus* Paperna, 1960 [30]) and only one species of *Cichlidogyrus* has been reported from a representative of *Orthochromis* (*C. consobrini* Jorissen, Pariselle & Vanhove 2018 [29]). However, this is likely an underestimation of the true species richness of this genus on these hosts. Indeed, taxonomic reports of *Cichlidogyrus* often indicate that the diversity on a single host can reach high levels, e.g. 25 species of *Cichlidogyrus* have been reported from the host *Coptodon guineensis* (Günther) [18].

## Methods

### Collection, sample preparation and conservation

**Fig. 1:**
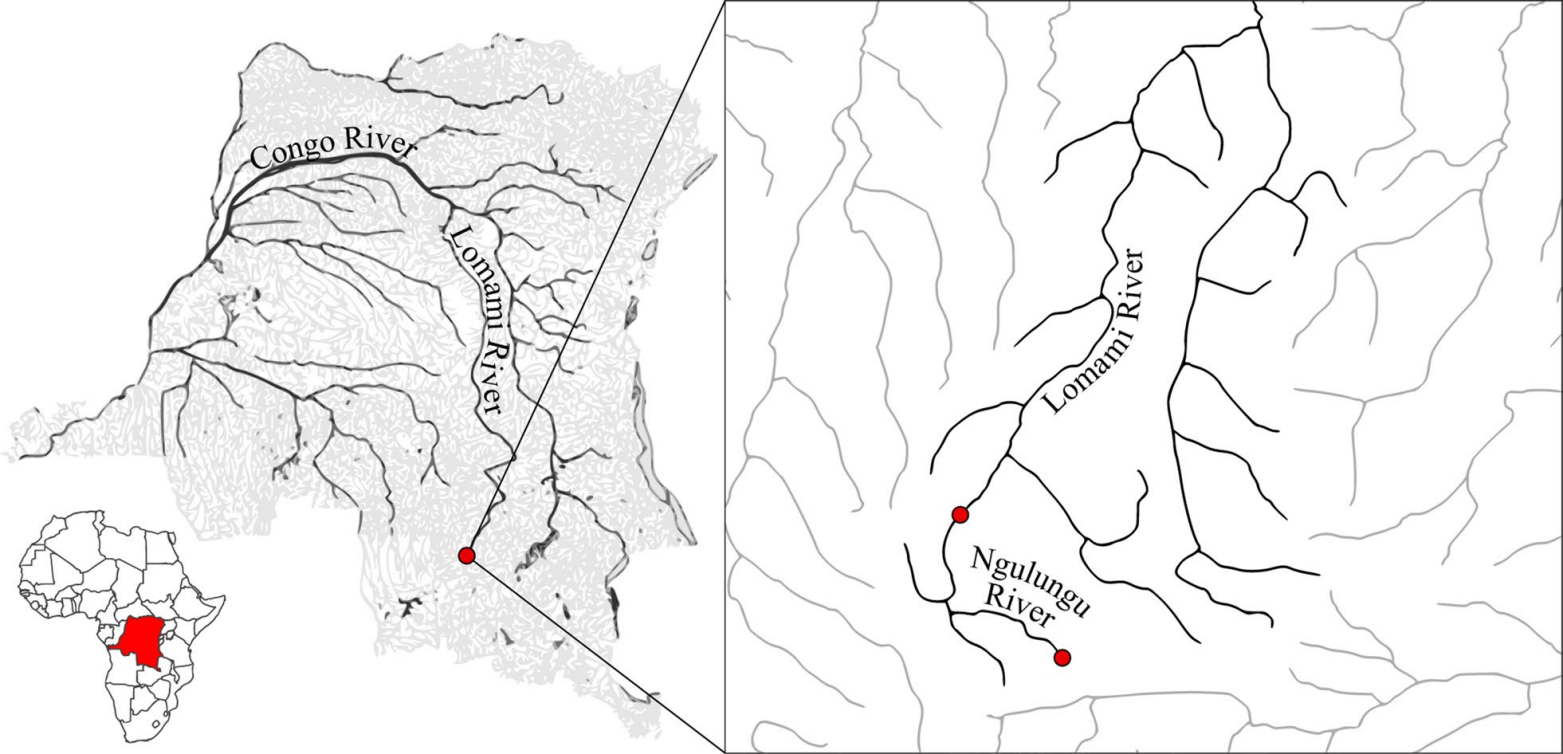
Map of the DRC situated on the African continent (left) and sampling region (right). Rivers in grey with in black the main streams (left) and Lomami Basin (right). Sampling locations are indicated as red dots (shapefiles downloaded from the Digital Chart of the World database using DIVA-GIS, maps created using QGis 2.18.14 software)

Fish were bought from local fishermen from the Upper Lomami Basin in the Haut-Lomami province (DRC), during a field expedition in May 2017. Specimens of *Serranochromis* cf. *macrocephalus* and *Orthochromis* sp. ‘Lomami’ were caught in the main stream (8°33′36″S, 24°36′36″E) on 29–30 May 2017. Specimens of *Tilapia sparrmanii* were captured from the Ngulungu River (8°44′09″S, 24°43′58″E), an affluent of the Lomami River, on 22 May 2017 (Fig. 1). The specimens of *Orthochromis* sp. ‘Lomami’ were caught using dip nets in the rapids of the river. Specimens of *Serranochromis* cf. *macrocephalus* were caught using seine nets. Specimens of *Tilapia sparrmanii* were caught using a combination of dip nets and fish pots. Fish were fixed in the field with formaldehyde (10%). Gills of the right gill chamber were dissected in the laboratory and screened exhaustively for monogenean parasites using an entomological needle under an Optica 4.0.0 stereomicroscope. Parasites were mounted on glass slides under a coverslip, directly in a drop of Hoyer’s medium. Coverslips were sealed with nailpolish.

Fish were deposited in the ichthyology collection at the Royal Museum of Central Africa in Tervuren (Belgium) and stored in 70% ethanol. Specimens of *Serranochromis* cf. *macrocephalus* are stored under the collection numbers RMCA_Vert_2017.018.P.0019–0021. Specimens of *Tilapia sparrmanii* are stored under the collection numbers RMCA_Vert_2017.018.P.0022–0029. Specimens of *Orthochromis* sp. ‘Lomami’ are stored under the collection numbers RMCA_Vert_2017.018.P.0001 and RMCA_Vert_2017.018.P.0004–0005. Mounted parasite specimens were deposited in the invertebrate collection of the Royal Museum of Central Africa, Tervuren, Belgium (RMCA); the collection of the research group Zoology: Biodiversity and Toxicology at Hasselt University, Diepenbeek, Belgium (HU); the Finnish Museum of Natural History, Helsinki, Finland (MZH); and the Iziko South African Museum, Cape Town, Republic of South Africa (SAMC) (see “Type-material” for details on repositories and accession numbers).

### Microscopy and illustrations

**Fig. 2:**
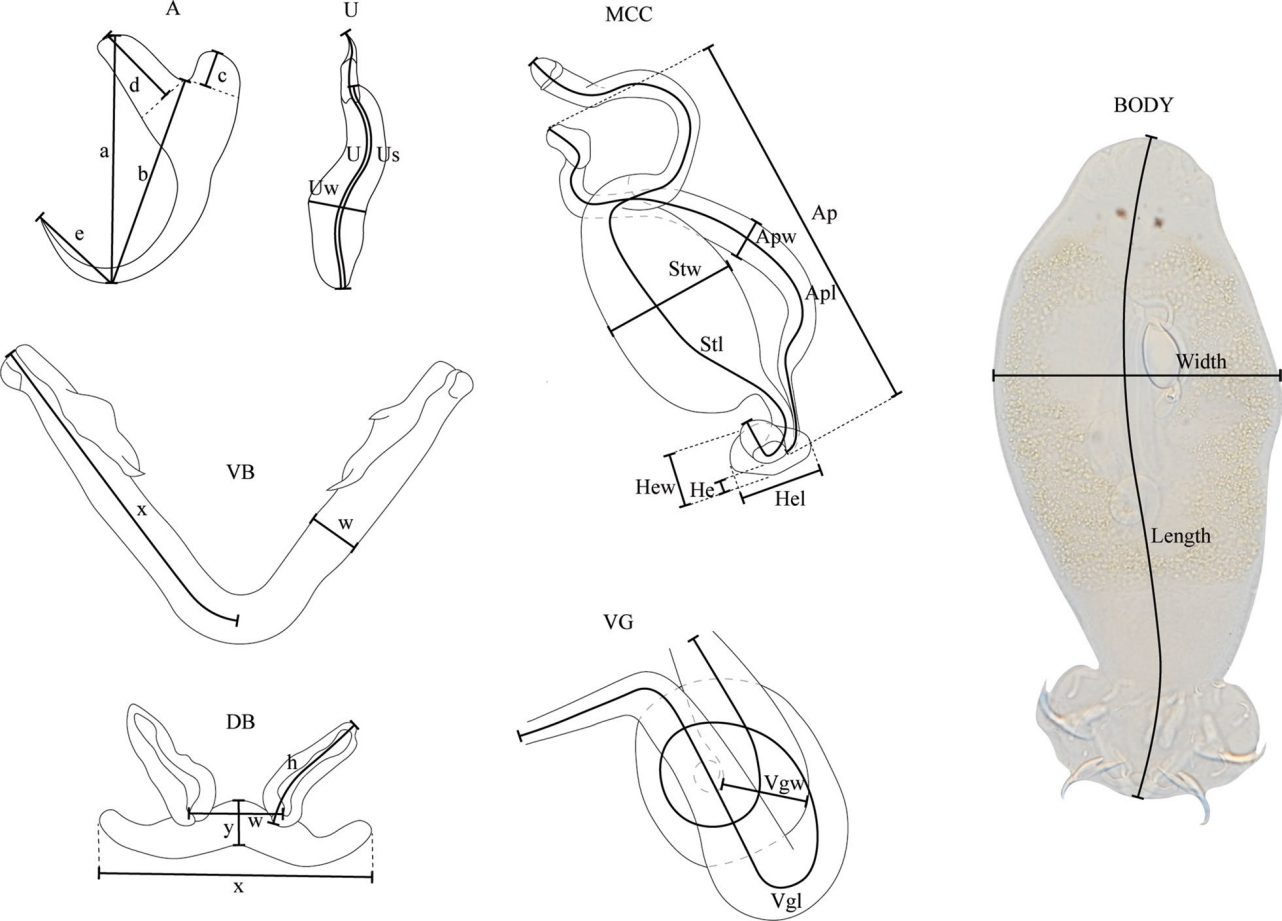
Measurements used in the description of the new species of *Cichlidogyrus* (measurements on the MCC and vagina are based on those of *C. bulbophallus* n. sp.). *Abbreviations*: A, anchors (a, anchor total length; b, anchor blade length; c, anchor shaft length; d, anchor guard length; e, anchor point length); U, uncinuli (U, total axial length; Us, axial length of secondary shaft; Uw, maximum width of first uncinuli); VB, ventral transverse bar (w, ventral bar maximum width; x, axial length of one branch); DB, dorsal transverse bar (h, axial length of the dorsal bar auricle; w, dorsal bar maximum width; y, distance between auricles; x, dorsal bar total length); MCC, male copulatory complex (Stl, axial length of penis stylet; Stw, maximum width of the penis stylet; Apl, axial length of accessory piece; Ap, linear length of the accessory piece; Apw, maximum width of the accessory piece; He, partial length of the heel; Hel, total length of the heel; Hew, width of the heel); VG, vagina (Vgl, total axial length; Vgw, maximum width), BODY, body

As dactylogyrid monogeneans are often differentiated based on their hard parts [21] and because soft internal organs were no longer visible in the whole-mounted specimens after fixation, the species descriptions focus on the details of the sclerotised parts, i.e. haptor, male copulatory complex (MCC) and vagina (when sclerotised). These hard parts were studied on the whole-mounted specimens with a Nikon Eclipse 80i compound microscope using interference contrast. Measurements were based on those of Pariselle & Euzet [32]. Additional measurements were taken for the uncinuli and the MCC (Table 1, Fig. 2). In total, 42 different metrical features were measured. For each species, the average, the range and number of measured specimens are provided. Measurements and drawings were made with the aid of a drawing tube at a magnification of 1000× (objective ×100 immersion, ocular ×10). Drawings were made freehand and edited in Adobe Illustrator CS5.1. Drawings are from multiple specimens in case not all structures were clearly visible in one single specimen. Micrographs were also taken with the Nikon Eclipse 80i and processed using NIS-Elements. Filaments associated with anchors and uncinuli are not presented as they are not diagnostically informative.

**Table 1:** Additional measurements to those made in Pariselle & Euzet [32]

Structure	Measurement	Definition
Accessory piece	Apl	Total axial length
	Apw	Maximum width
Penis stylet	Stw	Maximum width
Heel	Hel	Maximum length
	Hew	Maximum width
Uncinuli	Us	Length of secondary shaft
	Uw	Maximum width of first uncinuli

In literature, several terms are used to indicate specific structures of the posterior adhesive organ of representatives of the Dactylogyridae: the posterior adhesive organ is called *opisthhaptor* or simply *haptor*, the large hooks are called *anchors* or *hamuli*, small marginal hooks are indicated by the term *hooklets* or *uncinuli*, and the crosspieces connecting the large hooks are called the *V-shaped*/*ventral* and *compound*/*dorsal supporting*/*connective*/*transverse* bars [3, 14, 16, 28, 33]. Throughout this paper, we follow the terminology proposed by Fannes et al. [34]. Uncinuli are numbered as in Pariselle & Euzet [32]. We refer to the penis or copulatory tube as *penis stylet* as it essentially consists of a sclerotised hard structure [33]. Species were identified to genus level following Paperna [14] and the identification key of Pariselle & Euzet [21]. Different species of *Cichlidogyrus* were first assigned to one of the four morphological groups according to the relative size of their haptoral sclerites [21]: one group having long uncinuli I and long uncinuli III to VII, a second group consisting of species with short uncinuli I and long uncinuli III to VII, a third group consisting of species with short uncinuli I and short uncinuli III to VII, and finally a fourth group including species with long uncinuli I and short uncinuli III to VII. Uncinuli were called long or short based on their standardised length i.e. the division of their total length by the total length of the second uncinuli, which retain their larval size [21].

For the identification of new species, the phylogenetic species concept was adopted which states that species are reproductively isolated groups of natural populations that originate through a speciation event and end with the next speciation or vanish through extinction [35]. Practically, this means that a group of specimens is defined as a new species when they consistently differ from another group of specimens in at least one attribute [36]. Note that the authors of the new species are different from the authors of this article (International Commission on Zoological Nomenclature 2015 [37]).

Museum specimens from the invertebrate collection of the Royal Museum of Central Africa, Tervuren, Belgium, were used for morphological comparison with the new species (voucher specimens of *C. zambezensis*: MT.37714, MT.38165–MT.38171, MT.37984–MT.37986, MT.37988–MT.37992; paratypes of *C. reversati* Pariselle & Euzet 2003: MT.37400–MT.37402; and a paratype of *C. legendrei* Pariselle & Euzet 2003: MT.37400).

To comply with the regulations set out in article 8.5 of the amended 2012 version of the *International Code of Zoological Nomenclature* (ICZN) [38], details of the new species have been submitted to ZooBank. The Life Science Identifier (LSID) of the article is urn:lsid:zoobank.org:pub:845B6B53-7ED0-4100-8051-CC8A47B3481B. For each new species, the Life Science Identifier (LSID) is reported in the taxonomic summary.

## Results

Three specimens of *S.* cf. *macrocephalus*, three of *Orthochromis* sp. ‘Lomami’, and eight of *T. sparrmanii* were sampled. A total of 171 monogeneans were found on these cichlid species: 132 specimens on *S.* cf. *macrocephalus*, 30 specimens on *T. sparrmanii* and 9 specimens on *Orthochromis* sp. ‘Lomami’. These all belong to six species of *Cichlidogyrus*, which are all new to science.

Species descriptions are presented below. Measurements of the hard parts are provided in Table 2.

**Table 2:** Measurements (in micrometres) for the six new species of *Cichlidogyrus* described in this study

Species	*C. bulbophallus* n. sp.	*C. pseudozambezensis* n. sp.	*C. flagellum* n. sp.	*C. maeander* n. sp.	*C. lobus* n. sp.	*C. ranula* n. sp.
Host	*Serranochromis* cf. *macrocephalus*		*Tilapia sparmanii* Smith, 1840			*Orthochromis* sp. ‛Lomamiʼ
Locality	Lomami River (8°33′36″S, 24°36′36″E)	Lomami River (8°33′36″S, 24°36′36″E)	Ngulungu River (8°44′9″S, 24°43′58″E)	Ngulungu River (8°44′09″S, 24°43′58″E)	Ngulungu River (8°44′9″S, 24°43′58″E)	Lomami River (8°33′36″S, 24°36′36″E)
Additional hosts	–	–	–	*Orthochromis* sp. ‛Lomamiʼ	–	*Serranochromis* cf. *macrocephalus*
Additional localities	–	–	–	Lomami River (8°33′36″S, 24°36′36″E) on *Orthochromis* ‛Lomamiʼ	–	–
Number of specimens	*n *= 66	*n *= 63	*n *= 8	*n *= 15	*n *= 5	*n *= 3
Ventral anchor						
Total length, *a*	52 (47–56, *n *= 45)	43 (37–47, *n *= 29)	31 (26–36, *n *= 6)	34 (33–36, *n *= 6)	38 (31–42, *n *= 5)	31–32 (*n *= 2)
Blade length, *b*	41 (32–46, *n *= 47)	36 (31–42, *n *= 28)	28 (26–29, *n *= 6)	28 (25–30, *n *= 7)	34 (32–37, *n *= 5)	31 (*n *= 2)
Shaft length, *c*	9 (6–12, *n *= 48)	6 (3–9, *n *= 30)	5 (4–6, *n *= 6)	6 (5–7, *n *= 8)	8 (6–9, *n *= 5)	4 (2–5, *n *= 3)
Guard length, *d*	20 (15–23, *n *= 47)	11 (6–18, *n *= 27)	9–10 (*n *= 6)	11 (7–13, *n *= 8)	19 (17–21, *n *= = 5)	7 (*n *= 3)
Point length, *e*	18 (12–23, *n *= 62)	13 (10–16, *n *= 45)	10 (9–11, *n *= 6)	12 (11–14, *n *= 14)	12 (11–13, *n *= 5)	11–12 (*n *= 3)
Dorsal anchor						
Total length, *a*	56 (48–66, *n *= 40)	43 (34–47, *n *= 27)	27 (24–30, *n *= 7)	43–44 (*n *= 2)	33–34 (*n *= 2)	25–26 (*n *= 2)
Blade length, *b*	34 (28–38, *n *= 39)	33 (24–39, *n *= 25)	19 (16–20, *n *= 7)	28 (25–32, *n *= 2)	27 (26–28, *n *= 2)	21 (20–23, *n *= 2)
Shaft length, *c*	9 (5–13, *n *= 41)	7 (4–12, *n *= 23)	5 (3–6, *n *= 7)	10 (7–15, *n *= 3)	11–12 (*n *= 4)	4 (*n *= 2)
Guard length, *d*	26 (22–32, *n *= 41)	14 (10–19, *n *= 24)	10 (8–12, *n *= 7)	21–22 (*n *= 2)	13–14 (*n *= 3)	8–9 (*n *= 2)
Point length, *e*	11 (7–13, *n *= 53)	10 (7–14, *n *= 41)	6–7 (*n *= 7)	8 (6–10, *n *= 12)	4–5 (*n *= 3)	7 (*n *= 2)
Ventral transverse bar						
Branch length, *x*	74 (62–86, *n *= 42)	50 (46–56, *n *= 25)	34 (30–37, *n *= 5),	49 (42–56, *n *= 8)	36 (35–40, *n* = 4)	45 (41–51, *n* = 3)
Maximum width, *w*	11 (9–15, *n* = 63)	9 (7–11, *n* = 48)	5 (3–6, *n* = 7)	7–8 (*n* = 15)	8–9 (*n* = 5)	6–7 (*n* = 3)
Dorsal transverse bar						
Total length, *x*	57 (44–66, *n* = 61)	48 (42–60, *n* = 46)	26 (23–31, *n* = 7)	43 (37–50, *n* = 15)	54 (51–58, *n* = 5)	33 (*n* = 3)
Distance between auricles, *y*	24 (17–34, *n* = 52)	19 (16–27, *n* = 43)	12 (*n* = 6)	20 (17–27, *n* = 12)	17 (14–19, *n* = 5)	13–14 (*n* = 2)
Maximum width, *w*	12 (8–16, *n* = 50)	10 (7–12, *n* = 42)	2–3 (*n* = 7)	9 (7–11, *n* = 14)	8 (7–10, *n* = 5)	5 (4–6, *n* = 3)
Auricle length, *h*	31 (25–39, *n* = 37)	25 (20–32, *n* = 34)	11 (8–12, *n* = 7)	18 (14–22, *n* = 12)	16 (12–20, *n* = 2)	22 (*n* = 1)
Uncinuli						
Length I, *U*_*I*_	51 (43–56, *n* = 52)	24 (19–28, *n* = 45)	11 (9–12, *n* = 5)	37 (32–41, *n* = 13)	25 (23–27, *n* = 5)	15 (*n* = 2)
Maximum width I, *Uw*	11 (8–14, *n* = 61)	2 (*n* = 46)	1 (*n* = 5)	8 (7–11, *n* = 14)	2 (*n* = 5)	1–2 (*n* = 3)
Length II, *U*_*II*_	13 (11–14, *n* = 42)	12 (9–14, *n* = 38)	11 (10–12, *n* = 4)	11 (10–12, *n* = 10)	16–17 (*n* = 3)	12–13 (*n* = 3)
Length III, *U*_*III*_	32 (28–37, *n* = 54)	23 (21–26, *n* = 22)	18 (16–21, *n* = 4)	21 (18–28, *n* = 13)	35 (34–37, *n* = 5)	35 (34–37, *n* = 3)
Length IV, *U*_*IV*_	34 (28–41, *n* = 45)	29 (26–32, *n* = 25)	26 (22–28, *n* = 6)	25 (22–33, *n* = 13)	32 (*n* = 5)	35 (32–37, *n* = 3)
Length V, *U*_*V*_	41 (36–45, *n* = 39)	30 (26–33, *n* = 39)	26 (22–30, *n* = 7)	30 (27–32, *n* = 14)	40 (38–42, *n* = 4)	34 (*n* = 1)
Length VI, *U*_*VI*_	39 (33–46, *n* = 51)	28 (24–33, *n* = 37)	22 (18–27, *n* = 7)	29 (26–32, *n* = 14)	36 (35–39, *n* = 4)	34 (32–37, *n* = 2)
Length VII, *U*_*VII*_	36 (31–42, *n* = 55)	27 (24–32, *n* = 40)	19 (16–22, *n* = 7)	26 (21–31, *n* = 12)	35 (32–37, *n* = 4)	33 (32–34, *n* = 2)
Length III-VII	36 (28–46, *n* = 244)	28 (21–33, *n* = 163)	22 (16–30, *n* = 31)	26 (18–33, *n* = 66)	35 (32–42, *n* = 22)	34 (32–37, *n* = 11)
Length secondary shaft I, *Us*_*I*_	41 (34–46, *n* = 52)	13 (9–17, *n* = 46)	–	27 (22–31, *n* = 13)	10 (9–11, *n* = 5)	2 (*n* = 2)
Length secondary shaft III, *Us*_*III*_	20 (17–23, *n* = 54)	12 (10–14, *n* = 22)	7 (5–10, *n* = 4)	9 (7–14, *n* = 13)	18 (16–20, *n* = 5)	22 (21–24, *n* = 3)
Length secondary shaft IV, *Us*_*IV*_	22 (16–26, *n* = 45)	16 (13–19, *n* = 25)	13 (9–15, *n* = 6)	12 (10–20, *n* = 13)	16 (12–25, *n* = 5)	20 (18–21, *n* = 3)
Length secondary shaft V, *Us*_*V*_	28 (23–33, *n* = 39)	19 (16–22, *n* = 39)	15 (11–18, *n* = 7)	18 (14–22, *n* = 14)	22 (20–25, *n* = 4)	21 (*n* = 1)
Length secondary shaft VI, *Us*_*VI*_	27 (23–32, *n* = 51)	17 (14–20, *n* = 36)	12 (10–13, *n* = 7)	18 (12–21, *n* = 14)	19 (17–21, *n* = 4)	21 (20–23, *n* = 2)
Length secondary shaft VII, *Us*_*VII*_	24 (19–29, *n* = 55)	16 (13–20, *n* = 40)	8 (7–10, *n* = 6)	14 (8–18, *n* = 12)	19 (18–22, *n* = 4)	21 (20–23, *n* = 2)
Length secondary shaft III-VII	24 (16–33, *n* = 243)	17 (10–22, *n* = 162)	11 (5–18, *n* = 30)	15 (7–22, *n* = 66)	19 (12–25, *n* = 22)	21 (20–24, *n* = 11)
MCC						
Length stylet, *Stl*	145 (118–159, *n* = 59)	60 (52–64, *n* = 62)	80 (72–85, *n* = 6)	30 (27–32, *n* = 13)	61 (57–64, *n* = 5)	38 (*n* = 2)
Maximum width stylet, *Stw*	29 (25–33, *n* = 63)	12 (9–15, *n* = 56)	5 (3–6, *n* = 8)	4 (3–5, *n* = 13)	2 (*n* = 5)	6–7 (*n* = 3)
Linear length accessory piece, *Ap*	77 (65–95, *n* = 64)	62 (55–71, *n* = 63)	40 (32–51, *n* = 8)	22 (16–28, *n* = 15)	35 (31– 39, *n* = 5)	32 (28–37, *n* = 3)
Axial length accessory piece, *Apl*	93 (76–110, *n* = 63)	66 (54–76, *n* = 62)	98 (77–121, *n* = 8)	47 (39–54, *n* = 15)	/	36 (34–38, *n* = 3)
Maximum width accessory piece, *Apw*	8 (7–10, *n* = 66)	10 (4–12, *n* = 61)	3–5 (*n* = 8)	4 (2–6, *n* = 15)	21 (18–23, *n* = 5)	5 (*n* = 3)
Length heel, *Hel*	12 (10–15, *n* = 60)	12 (8–15, *n* = 55)	5 (3–7, *n* = 6)	10 (9–11, *n* = 11)	11 (10–12, *n* = 5)	15 (*n* = 1)
Partial length heel, *He*	17 (14–20, *n* = 65)	1–2 (*n* = 63)	2 (*n* = 8)	5 (2–7, *n* = 14)	8 (7–9, *n* = 5)	6–7 (*n* = 3)
Width heel, *Hew*	17 (14–20, *n* = 65)	7 (6–11, *n* = 56)	4 (2–5, *n* = 7)	7 (6–10, *n* = 12)	15 (14–17, *n* = 5)	5 (*n* = 2)
Vagina						
Length, *Vgl*	219 (173–280, *n* = 60)	14 (12–16, *n* = 20)	62 (48–67, *n* = 6)	–	22–23 (*n* = 3)	13 (10–15, *n* = 3)
Maximum width, *Vgw*	22 (17–27, *n* = 64)	5 (3–7, *n* = 60)	4 (3–5, *n* = 8)	–	3–4 (*n* = 5)	4 (*n* = 3)
Body						
Length	463 (331–638, *n* = 63)	377 (223–491, *n* = 50)	267 (229–305, *n* = 6)	329 (223–381, *n* = 15)	328 (292–350, *n* = 5)	401 (310–496, *n* = 3)
Width	158 (100–240, *n* = 66)	90 (60–143, *n* = 63)	65 (41–87, *n* = 7)	86 (60–104, *n* = 15)	121 (110–138, *n* = 5)	126 (119–132, *n* = 3)

*Note*: Measurements are given as the mean followed by the range and number of specimens in parentheses

**Family Dactylogyridae Bychowski, 1933**

**Genus** ***Cichlidogyrus*** **Paperna, 1960**

***Cichlidogyrus bulbophallus*** **Geraerts & Muterezi Bukinga n. sp.**

***Type-host*:***Serranochromis* cf. *macrocephalus* (Perciformes: Cichlidae).

***Type-locality*:** Lomami River (8°33′36″S, 24°36′36″E), Democratic Republic of the Congo.

***Type-material*:** The holotype (RMCA 39079) and 4 paratypes (RMCA 39071, 39073) are deposited in the invertebrate collection of the Royal Museum of Central Africa, Tervuren, Belgium (RMCA); 44 paratypes (HU nos 622, 629, 631–635, 637–640, 645, 650, 654, 655, 659, 660, 662, 663, 666, 670, 678, 679, 681–684, 690–704, 706, 708) are deposited in the collection of the research group Zoology: Biodiversity and Toxicology at Hasselt University, Diepenbeek, Belgium (HU); 8 paratypes (MZH KN.13824-KN.13827, KN.13829, KN.13830, KN.13834, KN.13835) are deposited in the Finnish Museum of Natural History, Helsinki, Finland (MZH); and 9 paratypes (SAMC A091369-A091371) are deposited in the Iziko South African Museum, Cape Town, Republic of South Africa (SAMC).

***Site in host*:** Gills.

***Prevalence and intensity*:** In 3 out of 3 hosts studied. All three specimens of *S.* cf. *macrocephalus* harboured specimens of *C. bulbophallus* n. sp. with an infection intensity of 3, 22 and 41, respectively.

***ZooBank registration*:** The Life Science Identifier (LSID) for *Cichlidogyrus bulbophallus* Geraerts & Muterezi Bukinga n. sp. is urn:lsid:zoobank.org:act:6CEC158A-33DB-40B8-80B7-9907EABA291A.

***Etymology*:** The species epithet is a combination of the Latin words *bulbus* (m) (= bulb) and *phallus* (m) (= penis). The name refers to the bulb-shaped swollen part of the penis stylet.

**Description**

**Fig. 3:**
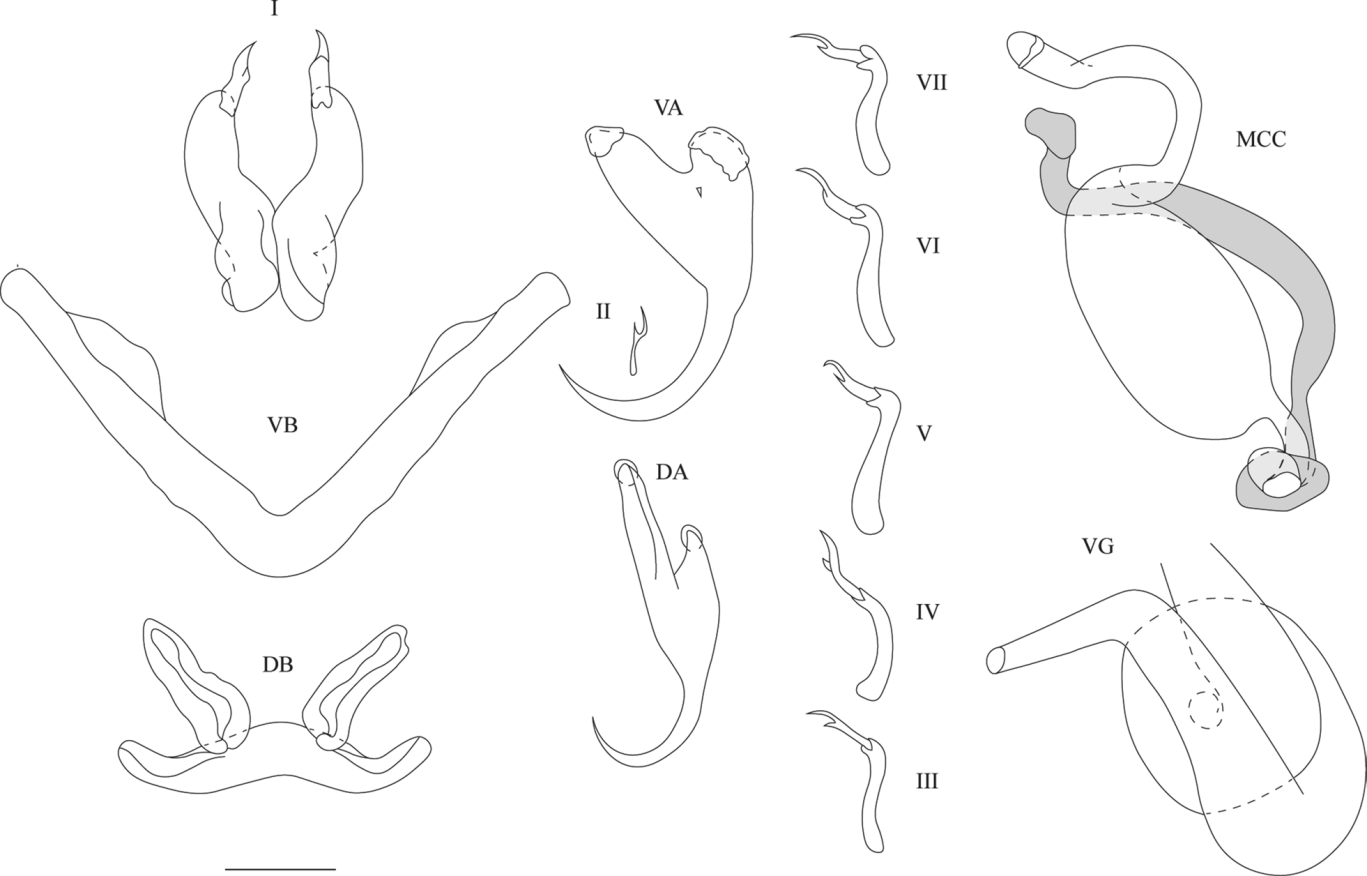
Sclerotised structures of *Cichlidogyrus bulbophallus* n. sp. Drawings based on five specimens: the holotype RMCA 39079 for the MCC and vagina; paratype HU no 633 for the VA and DB; paratype HU no 694 for the VB; paratype HU no 634 for the DA; and paratype HU no 635 for the uncinuli. *Abbreviations*: I-VII, uncinuli; VA, ventral anchor; VB, ventral transverse bar; DA, dorsal anchor; DB, dorsal transverse bar; MCC, male copulatory complex with penis stylet in white, and accessory piece and heel in grey; VG, vagina. *Scale-bar*: 20 µm

[Based on 66 specimens; metrical data in Table 2; see Fig. 3.] Anchors 2 pairs. Ventral anchors large, with guard approximately 2 times as long as shaft. Dorsal anchors of about same total length as ventral anchors; guard and shaft pronounced, asymmetrical; guard approximately 3 times as long as shaft. Blades of both ventral and dorsal anchors arched. Ventral transverse bar V-shaped, with 2 branches with wing-shaped attachments along distal half. Dorsal transverse bar made up of thick midsection, tapering towards its extremities, and 2 pronounced auricles inserted at its dorsal face. Uncinuli 7 pairs; uncinuli I long; uncinuli III to VII long.

MCC consisting of long penis stylet, accessory piece and heel. Proximal (18–33 µm, *n* = 61) and distal (46–78 µm, *n* = 66) ends of stylet tubiform, middle part (46–65 µm, *n* = 66) forming prominent bulb-shaped enlargement; stylet tube with approximately same diameter at proximal and distal ends. Base of penis stylet lacking distinct swollen bulb, attached to pronounced leaf-like heel. Accessory piece shorter than stylet, with a cap at distal end, connected to base of stylet proximally. Vagina coiled, long and wide, with two turns.

**Differential diagnosis**

The specimens display all diagnostic features of *Cichlidogyrus*: (i) two pairs of anchors (one dorsal and one ventral), two transverse bars (ventral bar V-shaped, dorsal bar with two auricles); (ii) 14 uncinuli; (iii) a male copulatory complex consisting of a penis stylet and usually an accessory piece; and (iv) a vagina which may or may not be sclerotised [14, 21]. *Cichlidogyrus bulbophallus* n. sp. shows an unusual haptor configuration: it has long uncinuli I and long uncinuli III to VII. Such combination has up to now only been described for five species: *C. arthracanthus* Paperna, 1960 [14], *C. inconsultans* Birgi & Lambert, 1987 [39, 40], *C. centesimus* Vanhove, Volckaert & Pariselle, 2011 [41], *C. calycinus* Kusters, Jorissen, Pariselle & Vanhove, 2018 (in some specimens) [20] and *C. habluetzeli* Rahmouni, Vanhove & Šimková, 2018 [19]. Based on the identification key, *Cichlidogyrus bulbophallus* n. sp. is similar to *C. inconsultans* (reported from *Polycentropsis abbreviata* Boulenger in Cameroon) [39]), *C. calycinus* (reported from *Hemichromis elongatus* (Guichenot) in the DRC) and *C. habluetzeli* (reported from *Cardiopharynx schoutedeni* Poll and *Cyphotilapia frontosa* (Boulenger) in the DRC) in having a sclerotised vagina (the vagina of *C. arthracanthus* and *C. centesimus* is not sclerotised). *Cichlidogyrus bulbophallus* n. sp. can be distinguished from these species, and any other species of *Cichlidogyrus*, based on the typical shape of its penis stylet, starting and ending as a tube of approximately the same diameter and with a distinct and greatly developed swollen middle portion. Also, the morphology of the vagina, which is long and wide, making two turns, is unique within the genus. Contrary to that of other species of *Cichlidogyrus*, the penis stylet of *C. bulbophallus* n. sp. does not show a distinctly swollen bulb at the base. This combination of characters has not yet been observed within *Cichlidogyrus*. Therefore, *C. bulbophallus* n. sp. can easily be recognised as a new species.

***Cichlidogyrus pseudozambezensis*** **Geraerts & Muterezi Bukinga n. sp.**

***Type-host*:***Serranochromis* cf. *macrocephalus* (Perciformes: Cichlidae).

***Type-locality*:** Lomami River (8°33′36″S, 24°36′36″E), Democratic Republic of the Congo.

***Type-material*:** The holotype (RMCA 39078) and 7 paratypes (RMCA 39072, 39074, 39078) are deposited in the invertebrate collection of the Royal Museum of Central Africa, Tervuren, Belgium (RMCA); 43 paratypes (HU nos 624–628, 630, 636, 642–644, 646–649, 651–653, 656–658, 661, 664, 667–669, 671–677, 680, 685–689, 705, 707, 709–712) are deposited in the collection of the research group Zoology: Biodiversity and Toxicology at Hasselt University, Diepenbeek, Belgium (HU); 6 paratypes (MZH KN.13828, KN.13831-KN.13833, KN.13836, KN.13837) are deposited in the Finnish Museum of Natural History, Helsinki, Finland (MZH); and 5 paratypes (SAMC A091372) are deposited in the Iziko South African Museum, Cape Town, Republic of South Africa (SAMC).

***Site in host*:** Gills.

***Prevalence and intensity*:** In 3 out of 3 hosts studied. All three specimens of *S.* cf. *macrocephalus* harboured specimens of *C. pseudozambezensis* n. sp. with an infection intensity of 2, 2 and 59, respectively.

***ZooBank registration*:** The Life Science Identifier (LSID) for *Cichlidogyrus pseudozambezensis* Geraerts & Muterezi Bukinga n. sp. is urn:lsid:zoobank.org:act:586511EB-98A5-4125-B867-0C741E432653.

***Etymology*:** The prefix *pseudo-* (Ancient Greek for ‘false, fake’) of the species epithet is used to emphasise the resemblance of the MCC to that of *C. zambezensis.*

**Description**

**Fig. 4:**
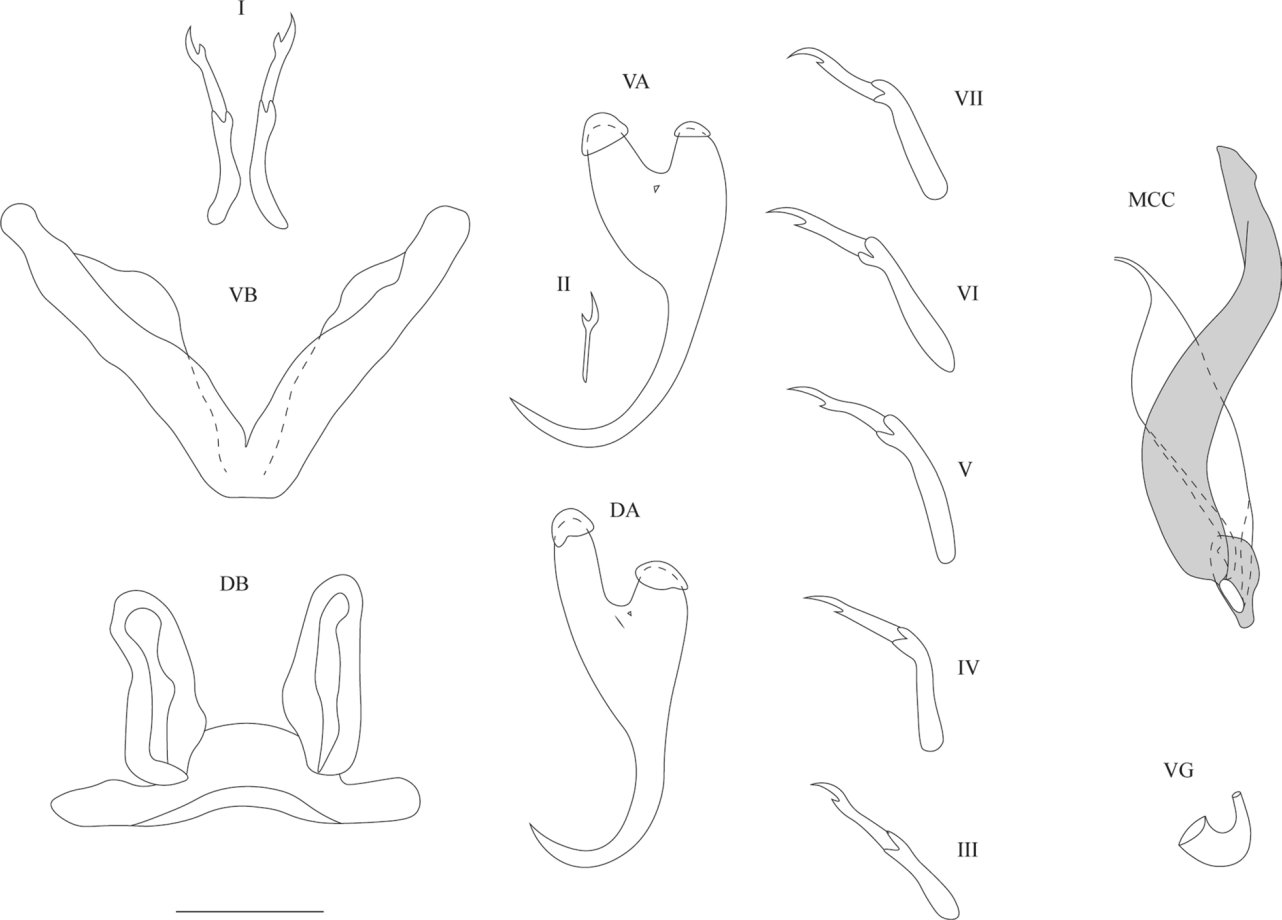
Sclerotised structures of *Cichlidogyrus pseudozambezensis* n. sp. Drawings are based on five specimens: the holotype RMCA 39078 for the vagina; paratype RMCA 39078 for the MCC; paratype HU no 627 for the uncinuli VA and DA; paratype HU no 651 for the DB; and paratype HU no 653 for the VB. *Abbreviations*: I-VII, uncinuli; VA, ventral anchor; VB, ventral transverse bar; DA, dorsal anchor; DB, dorsal transverse bar; MCC, male copulatory complex with penis stylet in white, and accessory piece and heel in grey; VG, vagina. *Scale-bar*: 20 µm

[Based on 63 specimens: metrical data in Table 2; see Fig. 4.] Anchors 2 pairs. Ventral anchors with guard slightly longer and more robust than shaft. Dorsal anchors of about same total length as ventral anchors; base more asymmetrical than that of ventral anchors; guard and shaft pronounced; guard approximately 2 times as long as shaft. Ventral transverse bar V-shaped with 2 branches with wing-shaped projections along distal half. Dorsal transverse bar made up of thick midsection, narrowing towards its extremities, and 2 pronounced auricles inserted at its dorsal face. Uncinuli 7 pairs; uncinuli I long, uncinuli IV to VII long, uncinuli III intermediate.

MCC consisting of penis stylet, accessory piece and heel. Penis stylet drop-shaped with pointed distal end and enlarged proximal basal bulb; midsection broadened. Base of penis stylet surrounded by irregular-shaped heel. Wall of penis stylet swollen at point where basal bulb attaches to heel. Accessory piece large and attached to base of stylet. Vagina C-shaped tube narrowing distally.

**Differential diagnosis**

The specimens display all diagnostic features of *Cichlidogyrus* (see “Differential diagnosis” for *C. bulbophallus* n. sp.). *Cichlidogyrus pseudozambezensis* n. sp. resembles *C. arthracanthus*, *C. inconsultans*, *C. centesimus*, *C. calycinus* and *C. habluetzeli* in having long uncinuli I and long uncinuli IV to VII [21, 22]. However, the MCC of *C. pseudozambezensis* n. sp. is morphologically similar to that of *C. zambezensis* (found on species of *Serranochromis* in Zimbabwe and the DRC, on *Oreochromis mortimeri* (Trewavas) in Zimbabwe [28], and on the *Sargochromis mellandi* (Boulenger) species complex in the DRC [29, 30]), a species that is positioned in a morphology-based group characterised by short uncinuli I and short uncinuli III to VII [21, 22]. *Cichlidogyrus pseudozambezensis* n. sp. also differs from *C. zambezensis* in the shape of the vagina, which is described as triangular, funnel-shaped or hat-like in *C. zambezensis* [28, 29]. Additionally, the accessory piece in *C. zambezensis* has a pointed distal end, in contrast to that in *C. pseudozambe*zensis n. sp., which distally ends in a more rounded tip.

***Cichlidogyrus flagellum*** **Geraerts & Muterezi Bukinga n. sp.**

***Type-host*:***Tilapia sparrmanii* Smith (Perciformes: Cichlidae).

***Type-locality*:** Ngulungu River (8°44′09″S, 24°43′58″E), Democratic Republic of the Congo.

***Type-material*** The holotype (RMCA 39075) is deposited in the invertebrate collection of the Royal Museum of Central Africa, Tervuren, Belgium (RMCA); 4 paratypes (H**:**U nos 615, 618, 619, 721) are deposited in the collection of the research group Zoology: Biodiversity and Toxicology at Hasselt University, Diepenbeek, Belgium (HU); 2 paratypes (MZH KN.13821, KN.13838) are deposited in the Finnish Museum of Natural History, Helsinki, Finland (MZH); and 1 paratype (SAMC A091368) is deposited in the Iziko South African Museum, Cape Town, Republic of South Africa (SAMC).

***Site in host*:** Gills.

***Prevalence and intensity*:** In 7 out of 8 hosts studied. Six specimens of *T. sparrmanii* harboured specimens of *C. flagellum* n. sp. with an infection intensity of 1, one with an infection intensity of 2.

***ZooBank registration*:** The Life Science Identifier (LSID) for *Cichlidogyrus flagellum* Geraerts & Muterezi Bukinga n. sp. is urn:lsid:zoobank.org:act:8C8DF873-A2DA-4819-8DE1-05C955E1F625.

***Etymology*:** The species epithet refers to the whip-like appearance of the penis stylet; *flagellum* (Lat., n) = whip.

**Description**

**Fig. 5:**
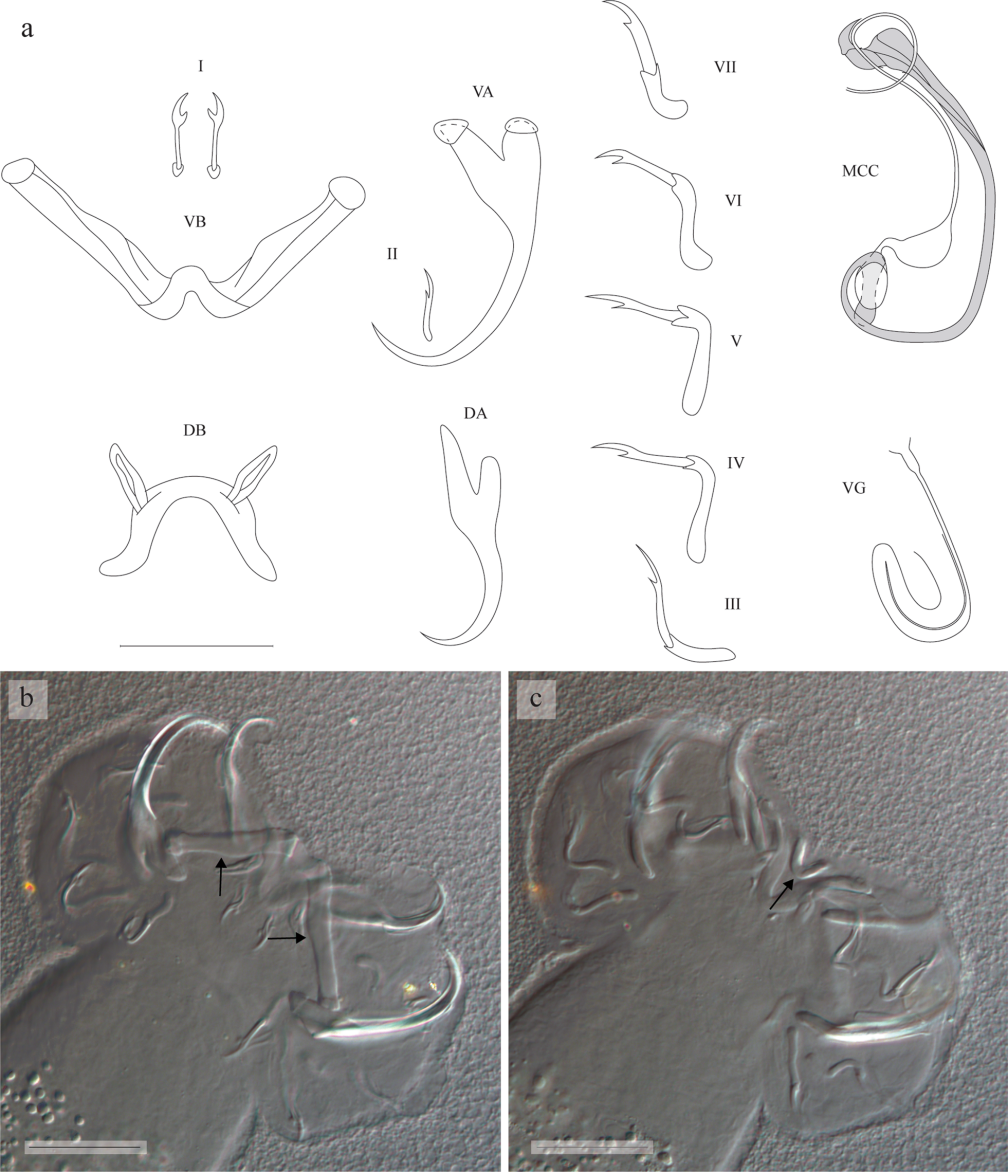
**a** Sclerotised structures of *Cichlidogyrus flagellum* n. sp. Drawings are based on four specimens: the holotype RMCA 39075 for uncinuli I, II, IV-VII and the vagina; paratype HU no 615 for uncinuli III; paratype MZH KN.13838 for the VB and MCC; and paratype HU no 721 for the VA, DA and DB. **b**, **c** Micrographs of the VB taken from paratype KN.13838 at two different focal depths. In (**b**), the two branches of the VB are indicated by an arrow. In (**c**), the middle part of the VB is indicated by an arrow. The VB looks W-shaped in the drawing (**a**) and micrographs (**b**, **c**) due to folding of this structure during the preparation of the microscope slide mount. *Abbreviations*: I-VII, uncinuli; VA, ventral anchor; VB, ventral transverse bar; DA, dorsal anchor; DB, dorsal transverse bar; MCC, male copulatory complex with penis stylet in white, and accessory piece and heel in grey; VG, vagina. *Scale-bars*: 20 µm

[Based on 8 specimens; metrical data in Table 2; see Fig. 5.] Anchors 2 pairs. Ventral anchors with guard approximately 2 times as long as shaft. Dorsal anchors somewhat shorter and less robust than ventral ones; guards, like those of ventral anchors, approximately 2 times as long as shafts. Ventral transverse bar V-shaped, with 2 branches with ridge along their length. Due to compression during preparation of microscope slides, ventral transverse bar not appearing V-shaped, but folded into other shapes. In paratype KN.13838 e.g., ventral transverse bar looking W-shaped (Fig. 5). Dorsal transverse bar more or less same width over its entire length; auricles short and inserted at dorsal face of bar. Uncinuli 7 pairs; uncinuli I short with short secondary shafts; uncinuli III and VII short; uncinuli IV, V, and VI long.

MCC consisting of penis stylet, accessory piece and heel. Penis stylet beginning as enlarged bulb, attached to small egg-shaped heel; penis stylet distinctly swollen at proximal third, proceeding distally as slender elongated whip-like tube, forming loop at distal end in all specimens. Accessory piece fish-hook-shaped, forming loop at beginning and joining penis stylet distally; large hook present at the end; accessory base proximally connected to base of penis stylet. Vagina sclerotised, folded and proximally broadened.

**Differential diagnosis**

The specimens display all diagnostic features of *Cichlidogyrus* (see “Differential diagnosis” for *C. bulbophallus* n. sp.). *Cichlidogyrus flagellum* n. sp. resembles *C. douellouae* Pariselle, Bilong Bilong & Euzet, 2003 (found on species of *Sarotherodon* Rüppell in West and West Central Africa [42]) in having short uncinuli I, long uncinuli IV to VI, a large vagina, a penis stylet length of more than 65 μm and an accessory piece ending in a hook. Clear differences between these two species can be found in the morphology of the reproductive organs. The vagina of *C. flagellum* n. sp. is longer (48–67 *vs* 12–17 µm in *C. douellouae*) and more curved than that of *C. douellouae.* The proximal third of the penis stylet of *C. flagellum* n. sp. is distinctly swollen and the penis stylet shows a loop near its distal end, in contrast to the penis stylet of *C. douellouae*, which does not have such swollen portion and loop. The accessory piece of *C. flagellum* n. sp. is fish-hook-shaped, while that of *C. douellouae* is S-shaped with a large and perpendicular diverticulum at its proximal third.

***Cichlidogyrus maeander*** **Geraerts & Muterezi Bukinga n. sp.**

***Type-host*:***Tilapia sparrmanii* (Perciformes: Cichlidae).

***Other host*:***Orthochromis* sp. ‘Lomami’ (Perciformes: Cichlidae).

***Type-locality*:** Ngulungu River (8°44′9″S, 24°43′58″E), the Democratic Republic of the Congo.

***Other locality*:** Lomami River (8°33′36″S, 24°36′36″E) on *Orthochromis* sp. ‘Lomami’, the Democratic Republic of the Congo.

***Type-material*:** The holotype (RMCA 39068) and 3 paratypes (RMCA 39068, 39077) are deposited in the Royal Museum of Central Africa, Tervuren, Belgium (RMCA); 7 paratypes (HU nos 614, 616, 620, 719, 722–724) are deposited in the collection of the research group Zoology: Biodiversity and Toxicology at Hasselt University, Diepenbeek, Belgium (HU); 3 paratypes (MZH KN.13822, KN.13840, KN.13841) are deposited in the Finnish Museum of Natural History, Helsinki, Finland (MZH); and 1 paratype (SAMC A091373) is deposited in the Iziko South African Museum, Cape Town, Republic of South Africa (SAMC).

***Site in host*:** Gills.

***Prevalence and intensity*:** In 6 out of 8 specimens of *T. sparrmanii* studied. Three specimens of *T. sparrmanii* were infected with an infection intensity of 3, two with an infection intensity of 2, and one with an infection intensity of 1. In 1 out of 3 specimens of *Orthochromis* sp. ‘Lomami’ studied. One specimen of *Orthochromis* sp. ‘Lomami’ was infected with an infection intensity of 1.

***ZooBank registration*:** The Life Science Identifier (LSID) for *Cichlidogyrus maeander* Geraerts & Muterezi Bukinga n. sp. is urn:lsid:zoobank.org:act:23713587-C596-46F3-983B-0B58F69E0E64.

***Etymology*:** The species epithet is deduced from Latin (*maeander* (m) = a road with a winding course) and refers to the sinusoid turning of the accessory piece around the penis stylet.

**Description**

**Fig. 6:**
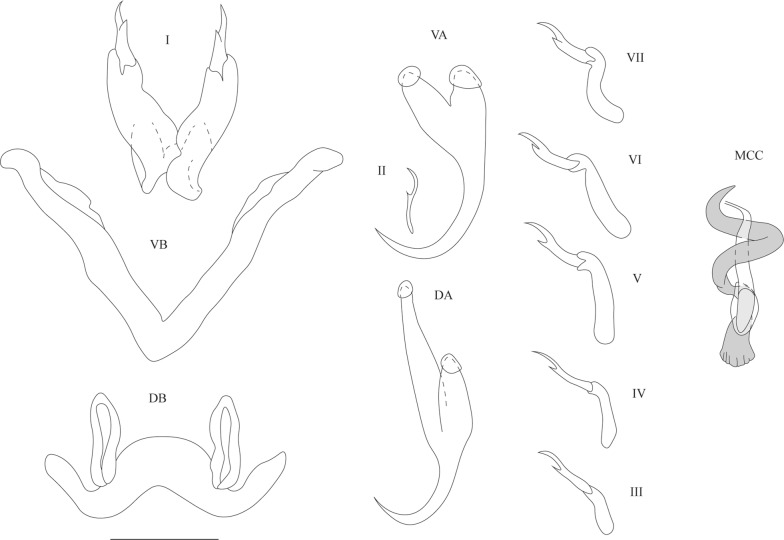
Sclerotised structures of *Cichlidogyrus maeander* n. sp. Drawings are based on three specimens: the holotype RMCA 39068 for the DA and MCC; paratype HU no 723 for the uncinuli, DB and VA; and paratype HU no 614 for the VB. *Abbreviations*: I-VII, uncinuli; VA, ventral anchor; VB, ventral transverse bar; DA, dorsal anchor; DB, dorsal transverse bar; MCC, male copulatory complex with penis stylet in white, and accessory piece and heel in grey. *Scale-bar*: 20 µm

[Based on 15 specimens; metrical data in Table 2; see Fig. 6.] Anchors 2 pairs. Ventral anchors with guard approximately 2 times as long as shaft. Dorsal anchors longer than ventral ones with longer shaft and guard; base consisting of pronounced pointed guard and blunt shaft, again with guard being approximately 2 times as long as shaft. Ventral transverse bar long and V-shaped with wing-like attachments along distal third of branches. Dorsal transverse bar arched with thick midsection, narrowing towards its extremities, with two pronounced auricles at its dorsal face. Uncinuli 7 pairs; uncinuli I long; uncinuli IV to VII long; uncinuli III of intermediate length.

MCC consisting of penis stylet, accessory piece and heel. Penis stylet short, forming enlarged bulb at base; base attached to pronounced heel; penis stylet distally curved, with pointed end. Accessory piece sinusoid and pointed at distal end; accessory piece narrowed proximally and attached to basal bulb of penis stylet. Vagina not observed.

**Differential diagnosis**

The specimens display all diagnostic features of *Cichlidogyrus* (see “Differential diagnosis” for *C. bulbophallus* n. sp.). *Cichlidogyrus maeander* n. sp. resembles *C. arthracanthus* and *C. centesimus*; all three of them have long uncinuli I and long uncinuli IV to VII, and lack a sclerotised vagina [14, 39, 40]. *Cichlidogyrus maeander* n. sp. can, however, easily be distinguished from both based on the shape of the MCC. The morphology of the MCC resembles more that of *C. reversati* (found on *Pelmatolapia cabrae* (Boulenger) in the mouth of the Bas Kouilou River (DRC) [32]). However, the distal end of the penis stylet of *C. maeander* n. sp. is pointed, while it is wide-mouthed in *C. reversati,* and the accessory piece of *C. maeander* is more pointed than that of *C. reversati*. Additionally, *C. maeander* n. sp. differs from *C. reversati* in the length of uncinuli III-VII (*C. reversati* having short uncinuli III-VII) [21]. Also, uncinuli I are more robust compared to those of *C. reversati*.

***Cichlidogyrus lobus*** **Geraerts & Muterezi Bukinga n. sp.**

***Type-host*:***Tilapia sparrmanii* (Perciformes: Cichlidae).

***Type-locality*:** Ngulungu River (8°44′9″S, 24°43′58″E), the Democratic Republic of the Congo.

***Type-material*:** The holotype (RMCA 39069) and 1 paratype (RMCA 39076) are deposited in the invertebrate collection of the Royal Museum of Central Africa, Tervuren, Belgium (RMCA); 1 paratype (HU no 617) is deposited in the collection of the research group Zoology: Biodiversity and Toxicology at Hasselt University, Diepenbeek, Belgium (HU); 1 paratype (MZH KN.13839) is deposited in the Finnish Museum of Natural History, Helsinki, Finland (MZH); and 1 paratype (SAMC A091373) is deposited in the Iziko South African Museum, Cape Town, Republic of South Africa (SAMC).

***Site in host*:** Gills.

***Prevalence and intensity*:** In 4 out of 8 hosts studied. One specimen of *T. sparrmanii* was infected with an infection intensity of 2 and three specimens were infected with an infection intensity of 1.

***ZooBank registration*:** The Life Science Identifier (LSID) for *Cichlidogyrus lobus* Geraerts & Muterezi Bukinga n. sp. is urn:lsid:zoobank.org:act:E6AB8EA6-1ED8-4972-8EBA-5FD5FAC371F7.

***Etymology*:** The species epithet refers to the lobed accessory piece; *lobus* (Lat.; m) = lobe.

**Description**

**Fig. 7:**
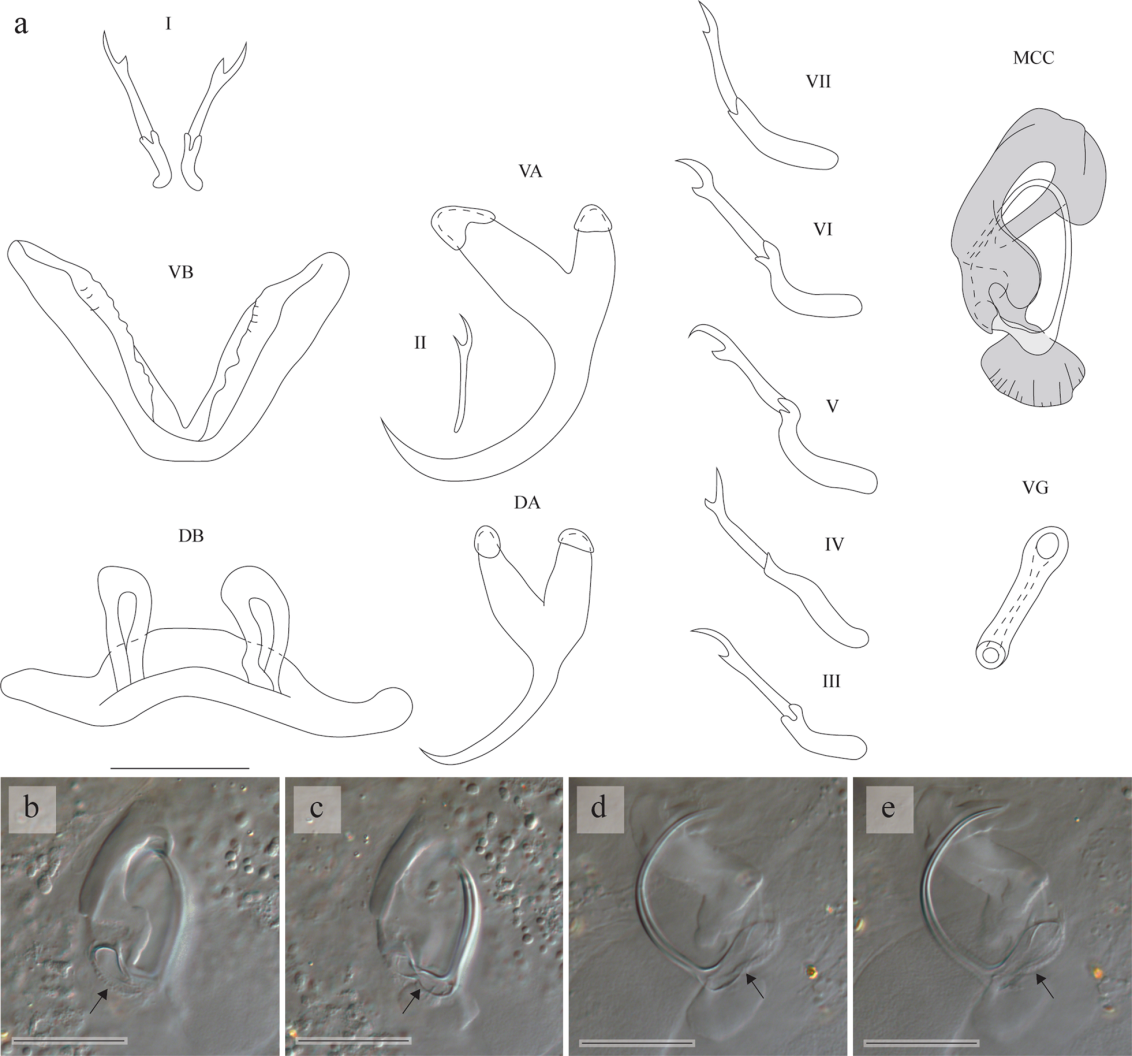
**a** Sclerotised structures of *Cichlidogyrus lobus* n. sp. Drawings are based on four specimens: the holotype RMCA 39069 for the MCC; paratype HU no 617 for the uncinuli and vagina; paratype SAMC A091373 for the VB and DB; and paratype MZH KN.13839 for the VA and DA. **b**–**e** Micrographs of the MCC of two specimens of *C. lobus* n. sp. **b**, **c** Micrographs taken from the holotype at two different focal depths. **d**, **e** Micrographs taken from paratype KN.13839 at two different focal depths. The arrows point at the basal bulb of the penis stylet. The penis stylet of the holotype seems to have a swollen portion distally from the basal bulb. This seemingly swollen portion is merely an artefact due to the folding of the basal bulb during preparation of the microscope slide mount. The basal bulb of paratype KN.13839 is not folded and shows that the penis stylet does not possess a swollen portion. *Abbreviations*: I-VII, uncinuli; VA, ventral anchor; VB, ventral transverse bar; DA, dorsal anchor; DB, dorsal transverse bar; MCC, male copulatory complex with penis stylet in white, and accessory piece and heel in grey; VG, vagina. *Scale-bars*: 20 µm

[Based on 5 specimens; metrical data in Table 2; see Fig. 7.] Anchors 2 pairs. Ventral anchors with robust base; guard approximately 2 times as long as shaft. Dorsal anchors approximately as long as ventral anchors, but thinner; base more symmetrical than that of ventral anchor; guard slightly longer than shaft; point slender and less curved compared to that of ventral one. Ventral transverse bar V-shaped with two robust branches with rough ridge over their entire length. Dorsal transverse bar made up of thick midsection, tapering towards its extremities, and 2 pronounced auricles inserted at its dorsal face. Uncinuli 7 pairs; uncinuli I short; uncinuli IV short; uncinuli III, V, VI, and VII long.

MCC consisting of penis stylet, accessory piece and heel. Penis stylet hook-shaped and pointed at distal end; base enlarged to ovoid basal bulb and attached to large heel. Penis stylet seeming to have swollen portion distally from basal bulb in holotype (Fig. 7), being merely an artefact due to folding of basal bulb during preparation of microscope slide mount. Accessory piece complex, consisting of several lobes; accessory piece proximally attached to basal bulb. Vagina short, thick-walled, and slightly arched in some specimens.

**Differential diagnosis**

The specimens display all diagnostic features of *Cichlidogyrus* (see “Differential diagnosis” for *C. bulbophallus* n. sp.). *Cichlidogyrus lobus* n. sp. resembles *C. legendrei* (found on *Pelmatolapia cabrae* in Lake Cayo (DRC) [32]). They can be distinguished from each other based on several details in the morphology of the haptor and MCC. Uncinuli IV of *C. lobus* n. sp. are short, while those of *C. legendrei* are long. The ventral transverse bar of *C. legendrei* has a slender appearance with two distinct wing-shaped appendages at the distal end of the branches. In contrast, the ventral transverse bar of *C. lobus* n. sp. is more robust with a rough ridge along the length of the branches; the wing-shaped appendages are absent. The penis stylet of *C. lobus* n. sp. is hook-shaped, in contrast to the sinuous penis stylet of *C. legendrei.* The pronounced hook at the end of the accessory piece of *C. legendrei* is not present in *C. lobus* n. sp.

***Cichlidogyrus ranula*** **Geraerts & Muterezi Bukinga n. sp.**

***Type-host*:***Orthochromis* sp. ‘Lomami’ (Perciformes: Cichlidae).

***Other host*:***Serranochromis* cf. *macrocephalus* (Perciformes: Cichlidae).

***Type-locality*:** Lomami River (8°33′36″S, 24°36′36″E), the Democratic Republic of the Congo.

***Type-material*:** The holotype (RMCA 39070) and 1 paratype (RMCA 39070) are deposited in the invertebrate collection of the Royal Museum of Central Africa, Tervuren, Belgium (RMCA); and 1 paratype (HU no 641) is deposited in the collection of the research group Zoology: Biodiversity and Toxicology at Hasselt University, Diepenbeek, Belgium (HU).

***Site in host*:** Gills.

***Prevalence and intensity*:** In 1 out of 3 specimens of *Orthochromis* sp. ‘Lomami’ studied; this specimen was infected with an infection intensity of 2. In 1 out of 3 specimens of *S.* cf. *macrocephalus* studied; this specimen was infected with an infection intensity of 1.

***ZooBank registration*:** The Life Science Identifier (LSID) for *Cichlidogyrus ranula* Geraerts & Muterezi Bukinga n. sp. is urn:lsid:zoobank.org:act:19CF0F3F-9971-4983-A637-87CBB303E0DE.

***Etymology*:** The species epithet refers to the tadpole-shaped penis stylet; *ranula* (Lat., f) = tadpole.

**Description**

**Fig. 8:**
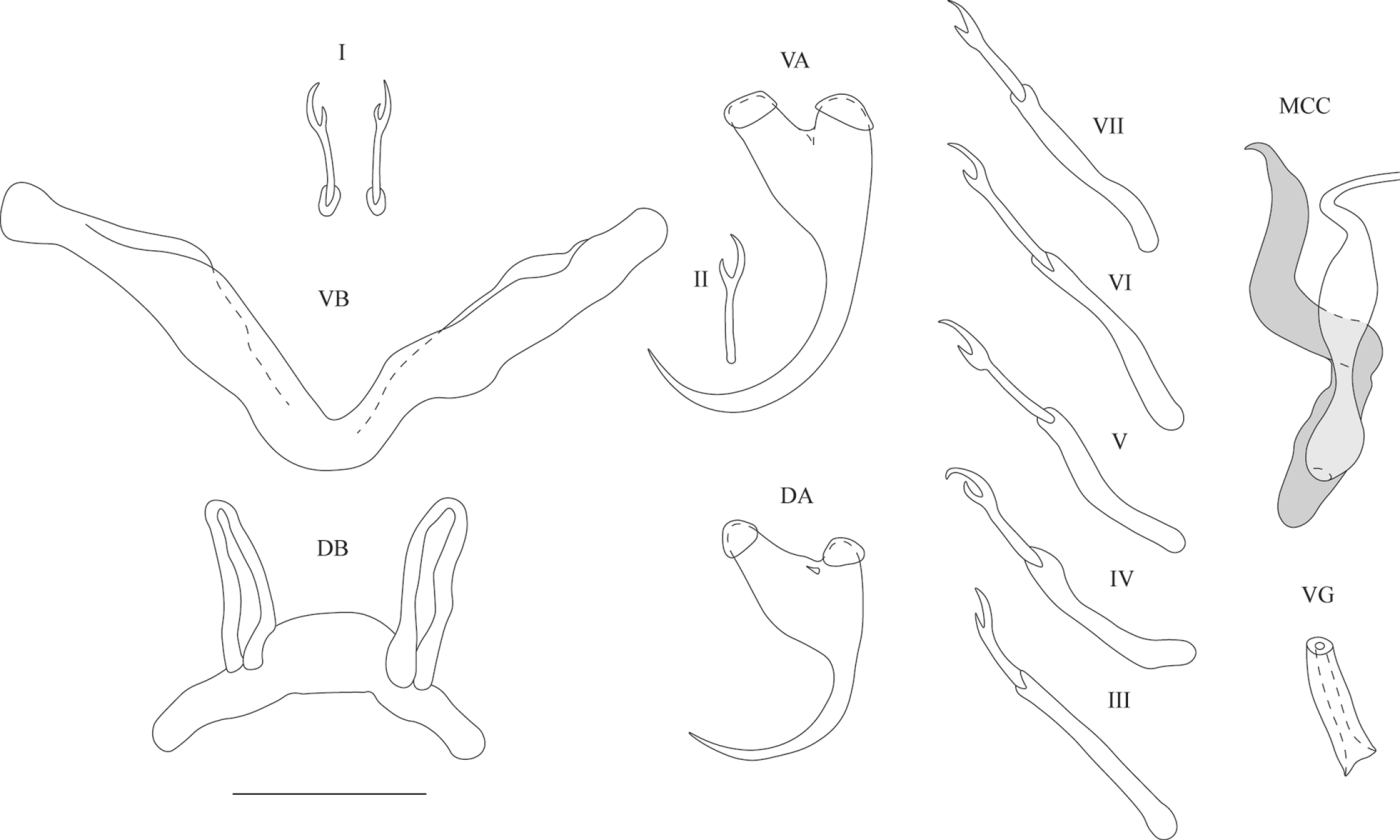
Sclerotised structures of *Cichlidogyrus ranula* n. sp. Drawings are based on three specimens: the holotype RMCA 39070 for the VA and MCC; paratype RMCA 39070 for the VB, DB, DA, uncinuli I and vagina; and paratype HU no 641 for uncinuli II-VII. *Abbreviations*: I-VII, uncinuli; VA, ventral anchor; VB, ventral transverse bar; DA, dorsal anchor; DB, dorsal transverse bar; MCC, male copulatory complex with penis stylet in white, and accessory piece and heel in grey; VG, vagina. *Scale-bar*: 20 µm

[Based on 3 specimens; metrical data in Table 2; see Fig. 8.] Anchors 2 pairs. Ventral anchors with robust asymmetric base; long guard approximately 2 times as long as shaft. Dorsal anchors slightly shorter than ventral ones; guard also approximately 2 times as long as shaft. Ventral transverse bar V-shaped with two long robust branches with wing-shaped attachments along distal third of each branch. Dorsal transverse bar made up of typically thick midsection, tapering towards its extremities, and two pronounced auricles at its dorsal face. Uncinuli 7 pairs; uncinuli I short with short secondary shafts; uncinuli III to VII long.

MCC consisting of penis stylet, accessory piece and heel. Penis stylet tadpole-shaped having swollen midsection and tapered, curved distal end; base enlarged to ovoid basal bulb and attached to long heel. Accessory piece extending from heel and having sinusoid course, ending distally in small hook. Vagina tube-shaped and thick-walled.

**Differential diagnosis**

The specimens display all diagnostic features of *Cichlidogyrus* (see “Differential diagnosis” for *C. bulbophallus* n. sp.). *C. ranula* n. sp. falls in the morphological group of species of *Cichlidogyrus* with short uncinuli I and long uncinuli III to VII. This species resembles *C. legendrei* and *C. lobus* n. sp. in having a short, rather straight, tubular vagina with a smooth wall. Clear differences with these two species are visible in the shape of the penis stylet and accessory piece: *C. legendrei* and *C. lobus* n. sp. have a penis stylet which lacks a broadened midsection (*vs* a broadened midsection in *C. ranula* n. sp.) and have a complex accessory piece (*vs* a simple accessory piece with a small hook at the end in *C. ranula* n. sp.). The MCC of *C. ranula* n. sp. shows some resemblance to that of *C. papernastrema* (found on *Tilapia sparrmanii* in South Africa and the DRC [29, 31]), which also has a penis stylet with a broadened midsection, tapering and curving towards the end, and an accessory piece ending in a hook. However, *C. papernastrema* lacks a sclerotised vagina and falls in a group of species showing a different haptoral construction, with long uncinuli I and short unicinuli III to VII.

### Remarks

Juveniles were identified by having a relatively large haptor compared to their body size, and no or not fully sclerotised copulatory organs. In total, twelve specimens of *Cichlidogyrus* were considered to be juveniles. In eight of these juveniles the copulatory organs were not yet developed: five of them occurring on *Orthochromis* sp. ‘Lomami’, the other three on *Tilapia sparrmanii.* The other four juveniles already possessed an MCC, though not yet fully sclerotised. These juveniles were not considered in the species descriptions and the calculation of the infection intensities.

## Discussion

### Monogenean species richness on the cichlid hosts

This present morphological survey revealed the presence of six new species of *Cichlidogyrus*: *C. bulbophallus* n. sp. and *C. pseudozambezensis* n. sp. found on *Serranochromis* cf. *macrocephalus*; *C. flagellum* n. sp. and *C. lobus* n. sp. on *Tilapia sparrmanii*; *C. ranula* n. sp. occurring on *Serranochromis* cf. *macrocephalus* and *Orthochromis* sp. ‘Lomami’; and *C. maeander* n. sp. found on *Orthochromis* sp. ‘Lomami’ and *Tilapia sparrmanii*.

Four species of *Cichlidogyrus* have previously been described from *T. sparrmanii*: *C. dossoui* [30]; *C. papernastrema* [29, 31]; *C. quaestio* [30]; and *C. tiberianus* [30]. *Cichlidogyrus papernastrema*, of which the MCC shows some resemblance with that of *C. ranula* n. sp., was described from the Zambezian Lowveld ecoregion (Natal, South Africa) [26, 31] and the Bangweulu-Mweru ecoregion (DRC) [26, 29, 31]. In the latter region, this species was also found on *Coptodon rendalli* (Boulenger) and *Oreochromis mweruensis* Trewavas.

Five species of *Cichlidogyrus* have been reported from *Serranochromis macrocephalus*: *C. dossoui* [28]; *C. halli* [28]; *C. quaestio* [28]; *C. sclerosus* [28]; and *C. zambezensis* [28, 29]. *Cichlidogyrus zambezensis*, of which the MCC shows some resemblance with that of *C. pseudozambezensis* n. sp., was originally described from the host *S. macrocephalus* from the Kariba Lake in Zimbabwe [28], where this species was also found to infect *Oreochromis mortimeri* [28]. In the Bangwuelu-Mweru ecoregion, *Cichlidogyrus zambezensis* was also found on *Serranochromis robustus jallae* (Günther), *S. stappersi* Trewavas, 1964, *S. angusticeps* (Boulenger), *S. thumbergi* (Castelnau) and the *Sargochromis mellandi* species complex [29, 30].

The only species of *Cichlidogyrus* described from *Orthochromis* spp. is *C. consobrini* on *Orthochromis katumbii* Schedel, Vreven, Manda, Abwe, Manda & Schliewen, 2018 from the Kiswishi River (DRC), where it was also found to infect the *Sargochromis mellandi* species complex [29, 43].

To sum up, after this study *Serranochromis* cf. *macrocephalus* (assuming it is a new fish species) and *Orthochromis* sp. ‘Lomami’ are known to harbour respectively three and two species of *Cichlidogyrus*, all described in this study. *Tilapia sparrmanii* is known to harbour seven species, of which three are described in the present study. The discovery of new species of *Cichlidogyrus* is not surprising, given the high species richness of this genus and the limited amount of studies reporting gill parasites from these hosts [21, 43].

### Morphological comparison with species from the Lower Guinea and Zambezi ichthyofaunal provinces

*Cichlidogyrus bulbophallus* n. sp., *C. pseudozambezensis* n. sp., *C. flagellum* n. sp. and *C. ranula* n. sp. all have a penis stylet with a swollen portion. Other congeners with such a distinct swollen portion are *C. amphoratus* Pariselle & Euzet, 1996 [44], *C. giostrai* Pariselle, Bilong Bilong & Euzet, 2003 [42], *C. njinei* Pariselle, Bilong Bilong & Euzet, 2003 [42], *C. ornatus* Pariselle & Euzet, 1996 [44], *C. papernastrema* [29, 31], *C. sanjeani* Pariselle & Euzet, 1997 [45] and *C. zambezensis* [28, 29]. These species are mainly reported from ichthyofaunal provinces in West Africa and West Central Africa (Upper Guinea province and Lower Guinea province) and Central Africa (Congo province and Zambezi province), all occurring on cichlids from the haplotilapiine lineage (Table 3) [12, 23, 24].

**Table 3:** Species of *Cichlidogyrus* with a distinct swollen portion in the penis stylet

Species	Host species	Location	Reference
*Cichlidogyrus amphoratus* Pariselle & Euzet, 1996	*Coptodon louka* (Thys van den Audenaerde)^a^	Bourouma River (Guinea)^a^	[44]
*Cichlidogyrus giostrai* Pariselle, Bilong Bilong & Euzet, 2003	*Sarotherodon caudomarginatus* (Boulenger)^a^	Badi River (Guinea)^a^	[42]
		Bourouma River (Guinea)	[42]
*Cichlidogyrus njinei* Pariselle, Bilong Bilong & Euzet, 2003	*Sarotherodon galilaeus* (L.)^a^	Sanaga River (Cameroon)^a^	[42]
*Cichlidogyrus ornatus* Pariselle & Euzet, 1996	*Coptodon zillii* (Gervais)^a^	Baoulé River (Ivory Coast)^a^	[44]
		Bagoué River (Ivory Coast)	[44]
	*Coptodon dageti* (Thys van den Audenaerde)	Comoé River (Ivory Coast)	[44]
*Cichlidogyrus papernastrema* Price, Peebles & Bamford, 1969	*Tilapia sparrmanii* Smith^a^	Natal (South Africa)^a^	[31]
		Bangwuelu-Mweru Ecoregion (DRC)	[29]
	*Coptodon rendalli* (Boulenger)	Bangwuelu-Mweru Ecoregion (DRC)	[29]
	*Oreochromis mweruensis* Trewavas	Bangwuelu-Mweru Ecoregion (DRC)	[29]
*Cichlidogyrus sanjeani* Pariselle & Euzet, 1997	*Sarotherodon occidentalis* (Daget)^a^	Bourouma River (Guinea)^a^	[45]
		Batapon River (Guinea)	[45]
		Little Scarcies River (Sierra Leone)	[45]
*Cichlidogyrus zambezensis* Douëllou, 1993	*Serranochromis macrocephalus* (Boulenger)^a^	Kariba Lake (Zimbabwe)^a^	[28]
		Bangwuelu-Mweru Ecoregion (DRC)	[29]
	*Oreochromis mortimeri* (Trewavas)	Kariba Lake (Zimbabwe)^a^	[28]
	*Serranochromis robustus* (Günther)	Bangwuelu-Mweru Ecoregion (DRC)	[29]
	*Serranochromis stappersi* Trewavas	Bangwuelu-Mweru Ecoregion (DRC)	[29]
	*Serranochromis thumbergi* (Castelnau)	Bangwuelu-Mweru Ecoregion (DRC)	[29]
	*Serranochromis angusticeps* (Boulenger)	Bangwuelu-Mweru Ecoregion (DRC)	[29]
	*Sargochromis mellandi* (Boulenger)	Bangwuelu-Mweru Ecoregion (DRC)	[29]

^a^Indicates the type-host and type-locality

Some species described in this study also showed morphological similarities to species reported from the ichthyofaunal province of Lower Guinea (*C. legendrei* and *C. reversati*) and the Upper Congo-Zambezi province (*C. zambezensis* and *C. papernastrema*) (see above and Table 3).

The Lomami River belongs to the Congo ichthyofaunal province. The Lower Guinea and the Congo ichthyofaunal province share part of their fish fauna, possibly as a consequence of the historic capture of some Lower Congo rivers by rivers in the Lower Guinea province [12]. Similarly, the Congo province and the Zambezi province show ichthyofaunal similarities, probably due to the past connection between the Upper Zambezi and the Kasai Basin and between the Upper Zambezi and the Upper Congo [12, 27, 46]. The morphological similarities of the MCC of species of *Cichlidogyrus* from the Lomami River Basin to those of the Lower Guinea and Upper Congo-Zambezi could be explained by convergent evolution, or alternatively be explained by these past river connections, which would have allowed the colonisation of Lower Guinean and Zambezian fish fauna into the Congo province. It would be worthwhile to use genetic data to investigate whether these morphological similarities are due to convergent evolution, typical to the monogenean fauna of certain ichthyofaunal provinces or host species, or whether this occurrence pattern is rather an artefact due to a sampling bias towards these regions and cichlid lineage.

### Host specificity

Adopting the delimitation of host specificity used in Mendlová & Šimková [47] and considering the limited number of hosts and parasites studied, *C. bulbophallus* n. sp., *C. pseudozambezensis* n. sp., *C. flagellum* n. sp. and *C. lobus* n. sp. appear to be strict specialists, infecting only one host species. *Cichlidogyrus ranula* n. sp. seems to be an intermediate generalist, infecting non-congeneric cichlid species from the same tribe (i.e. Haplochromini). Finally, *C. maeander* n. sp. appears to be a generalist, infecting non-congeneric cichlid species of at least two different tribes (i.e. Tilapiini and Haplochromini) [24, 25].

Comparable to what was reported by Jorissen et al. [29] for the Bangwuelu-Mweru ecoregion (Upper Congo Basin), the species of *Cichlidogyrus* found in the Lomami River (Middle Congo Basin) also range from strict specialists to generalists. The generalist *C. maeander* n. sp. infects both the substrate brooding *T. sparrmanii* as well as the mouthbrooding *Orthochromis* sp. ‘Lomami’. This is in line with the hypothesis that decreasing host specificity is associated with infecting fish hosts with each exhibiting a different form of parental care [47] and contradicts Pouyaud et al. [48], who stated that species of *Cichlidogyrus* are not able to infect hosts with different forms of parental care.

The parasites already described from *T. sparrmanii*, *S. macrocephalus* and *O. katumbii* are all intermediate generalists (*C. consobrini* and *C. halli*) or generalists (*C. dossoui*, *C. papernastrema*, *C. quaestio*, *C. sclerosus* and *C. zambezensis*) [29, 43]. This may suggest that the host range of the species of *Cichlidogyrus* that were described in this study as strict specialists may expand with the parasitological examination of more fish species. Therefore, additional sampling of more potential hosts at different locations is needed to confirm or reject the host specificity of the described parasite species because the host range of a parasite species may differ between different regions or considering different scales [29, 47, 49, 50]. A more thorough parasitological screening of the Lomami River and adjacent regions is also needed to answer the question whether the distribution pattern of these species of *Cichlidogyrus* is determined by the geography of the basin or rather mirrors the host biogeography [30].

### Perspectives

Species were initially identified following Paperna [14] and the identification key of Pariselle & Euzet [21]. The key of Pariselle & Euzet starts by splitting up the species of *Cichlidogyrus* into four haptoral groups (overview: Vignon et al. [22]). This split is based on the relative length of the uncinuli, considering pair III to VII of approximately the same relative length. This division into morphological groups was confirmed by Vignon et al. [22] and Pouyaud et al. [48], using molecular phylogenetic analyses based on *18S* rDNA and ITS1 sequences. They found that the morphological groups are partially congruent with the monophyletic groups obtained with the molecular phylogeny. In this study, however, some species of *Cichlidogyrus* (*C. pseudozambezensis* n. sp., *C. flagellum* n. sp., *C. maeander* n. sp. and *C. lobus* n. sp.) were found to have uncinuli pairs III to VII of different relative length. Rahmouni et al. [51] also found several species of *Cichlidogyrus* from Lake Tanganyika which do not fit into the previously reported classification and suggested the existence of more than four haptoral groups. Because many new species are being described, some with a different haptoral anatomy than previously known, revision of the current classification is greatly needed.

Nowadays, a genetic approach is an integral part of taxonomic studies. Genetic analyses can be used to distinguish cryptic species and to reveal the existence of phenotypic plasticity within a species and the convergent evolution of the shape and dimensions of the hard parts within *Cichlidogyrus* [48, 52, 53]. Regrettably, material for such molecular analyses was not available for the current study due to fixation with formaldehyde (see Methods).

## Conclusions

Sampling of fish in the Lomami River (DRC) revealed several species of Monogenea on the fish species *Serranochromis* cf. *macrocephalus*, *Orthochromis* sp. ‘Lomami’ and *Tilapia sparrmanii.* A total of six new species of *Cichlidogyrus* were found, ranging from strict specialists to generalists. The number of species of *Cichlidogyrus* possibly infesting *Serranochromis* cf. *macrocephalus*, *T. sparrmanii* and *Orthochromis* sp. ‘Lomami’ is raised substantially by the present study. *Serranochromis* cf. *macrocephalus* and *Orthochromis* sp. ‘Lomami’ are now known to harbour respectively three and two species of *Cichlidogyrus*, all described in this study. *Tilapia sparrmanii* are now known to harbour seven species, of which three are described in the present study. These results highlight the species diversity of *Cichlidogyrus* in the Congo Basin. The species described in this study show morphological similarities to species reported both from haplotilapiine fish from the Lower Guinea province and the Upper Congo-Zambezi province. Future research and additional sampling are needed to investigate whether these morphological similarities are due to convergent evolution, typical for the monogenean fauna of certain ichthyofaunal provinces or host species, or whether this occurrence pattern is rather an artefact due to a sampling bias towards these regions and cichlid lineage.

## Data Availability

The data supporting the findings of this article are included within the article. The type-material of the new species described in this study is deposited in the invertebrate collection of the Royal Museum of Central Africa, Tervuren, Belgium; the collection of the research group Zoology: Biodiversity and Toxicology at Hasselt University, Diepenbeek, Belgium; the Finnish Museum of Natural History, Helsinki, Finland; and the Iziko South African Museum, Cape Town, Republic of South Africa (see “Type-material” for details on repositories and accession numbers). Tissue samples of the fish hosts are available in the ichthyology collection of the Royal Museum of Central Africa, Tervuren, Belgium.

## Abbreviation

MCC: male copulatory complex
